# Chronic unpredictable stress influenced the behavioral but not the neurodegenerative impact of paraquat

**DOI:** 10.1016/j.ynstr.2019.100179

**Published:** 2019-05-31

**Authors:** Chris Rudyk, Zach Dwyer, Jessica McNeill, Natalina Salmaso, Kyle Farmer, Natalie Prowse, Shawn Hayley

**Affiliations:** a Department of Neuroscience, Carleton University, 1125 Colonel By Drive, Ottawa, Ontario, Canada, K1S 5B6; b Department of Neurology, University of Pittsburgh, Pittsburgh, PA, 15213, USA

**Keywords:** Parkinson's, Stress, Inflammatory, Microglia, Cytokine, Toxicity, AAR, alternate arm return, ANOVA, analysis of variance, BCA, bicinchoninic acid, BDNF, brain derived neurotrophic factor, CUS, chronic unpredictable stress, EDTA, ethylenediaminetetraacetic acid, ELISA, enzyme-linked immunosorbent assay, EPM, elevated plus maze, FST, forced swim test, GR, glucocorticoid receptor, HPA, hypothalamus-pituitary adrenal, IBA1, ionized calcium-binding adapter molecule 1, MMx, Micromax, PB, phosphate buffer, PBS, phosphate buffered saline, PD, Parkinson's disease, PFA, paraformaldehyde, pGR, phosphate glucocorticoid receptor, PVDF, polyvinylidene difluoride, RIPA, Radio Immuno Precipitation Assay, RR, rotarod, SAB, spontaneous alternation behavior, SAR, same arm return, SDS, sodium dodecyl sulphate, SNc, substantia nigra pars compacta, SPT, sucrose preference test, TH, tyrosine hydroxylase, VTA, ventral tegmental area

## Abstract

The impact of psychological stressors on the progression of motor and non-motor disturbances observed in Parkinson's disease (PD) has received little attention. Given that PD likely results from many different environmental “hits”, we were interested in whether a chronic unpredictable stressor regimen would act additively or possibly even synergistically to augment the impact of the toxicant, paraquat, which has previously been linked to PD. Our findings support the contention that paraquat itself acted as a systemic stressor, with the pesticide increasing plasma corticosterone, as well as altering glucocorticoid receptor (GR) expression in the hippocampus. Furthermore, stressed mice that also received paraquat displayed synergistic motor coordination impairment on a rotarod test and augmented signs of anhedonia (sucrose preference test). The individual stressor and paraquat treatments also caused a range of non-motor (e.g. open field, Y and plus mazes) deficits, but there were no signs of an interaction (neither additive nor synergistic) between the insults. Similarly, paraquat caused the expected loss of substantia nigra dopamine neurons and microglial activation, but this effect was not further influenced by the chronic stressor. Taken together, these results indicate that paraquat has many effects comparable to that of a more traditional stressor and that at least some behavioral measures (i.e. sucrose preference and rotarod) are augmented by the combined pesticide and stress treatments. Thus, although psychological stressors might not necessarily increase the neurodegenerative effects of the toxicant exposure, they may promote co-morbid behaviors pathology.

## Highlights

• Paraquat induced behavioral and neurochemical alterations similar to those induced by a chronic unpredictable stressor.
• Chronic unpredictable stress did not influence the degeneration of midbrain dopamine neurons or microglia activation.
• The paraquat and chronic stressor exposure resulted in augmented motor impairment and anhedonic-like behavior.

## 1 Introduction

Primarily characterized by the loss of dopamine neurons in the substantia nigra pars compacta (SNc), Parkinson's disease (PD) is the second most common neurodegenerative disease. Current evidence suggests that PD pathogenesis involves a complex interaction between a number of risk factors including genetic susceptibility, age, and environmental factors (i.e. exposure to psychological stress and/or exposure to chemical stressors like pesticides) that give rise to sporadic forms of the disease (Cannon and Greenamyre, 2013; Rappold et al., 2011). The link between PD and cumulative lifetime pesticide exposure has been supported for some time (Ritz et al., 2016; Wang et al., 2011), with studies showing chronically exposed plantation and farm workers having an increase likelihood of developing the disease (Moisan et al., 2015; Tanner et al., 2009). In particular, evidence suggests that the pro-oxidant herbicide, paraquat, can provoke neurodegeneration and other PD associated biological alterations (i.e. oxidative stress, pro-inflammatory milieu, Lewy-body like α–synuclein dense aggregates) within the nigrostriatal system (Mangano et al., 2012; Manning-Bog et al., 2002; McCarthy et al., 2004; McDowell and Chesselet, 2012; Shrestha et al., 2017; Wu et al., 2005). Recent evidence also suggests that the herbicide may be particularly germane for some of the non-motor features of the disease including select neuropsychiatric disturbances (i.e. anxiety and depression), olfactory dysfunction, as well as cognitive deficits (Ait-Bali et al., 2016; Chen et al., 2012; Czerniczyniec et al., 2011; Rudyk et al., 2017). Indeed, paraquat is widely distributed throughout the brain including the olfactory bulbs, prefrontal cortex, and hippocampus where it can impart a variety of neurochemical changes that may in part explain some of these behavioral deficits (Peng et al., 2007; Prasad et al., 2009; Rappold et al., 2011).

It is highly likely that paraquat acts in concert with a number of environmental factors in order to give rise to or augment the presentation PD symptoms (Ritz et al., 2016). It is now known that even psychologically relevant stressors may impact motor and non-motor manifestations seen in PD patients (Smith et al., 2002). For instance, major life events have been shown to impact the development of depression in patients (Rod et al., 2013) and patients report worsened tremor when in an anxious state (Smith et al., 2002). Most strikingly, chronic unpredictable stressor exposure accelerated the nigrostriatal damage and motor behavioral impairments induced by the PD relevant toxicants, MPTP or 6-OHDA, (Hemmerle et al., 2014; Janakiraman et al., 2017, 2016; Smith et al., 2008). Intriguingly, previous research from our lab has demonstrated that paraquat acts in a manner similar to chronic psychological stress in mice imparting hypothalamus-pituitary adrenal (HPA) axis activation, as well as inducing neurochemical alterations in a variety of stress sensitive brain regions (i.e. hypothalamus, hippocampus, central amygdala), supporting our contention that paraquat itself acts as a systemic stressor (Litteljohn et al., 2011; Rudyk et al., 2015). As such, we assessed the toxin's effects on a variety of non-motor behaviors (i.e. cognitive and neuropsychiatric) and associated neurochemical alterations together with exposure to a psychologically relevant chronic unpredictable stress regimen.

## 2 Methods

### 2.1 Animals and general experimental design

**Fig. 1:**
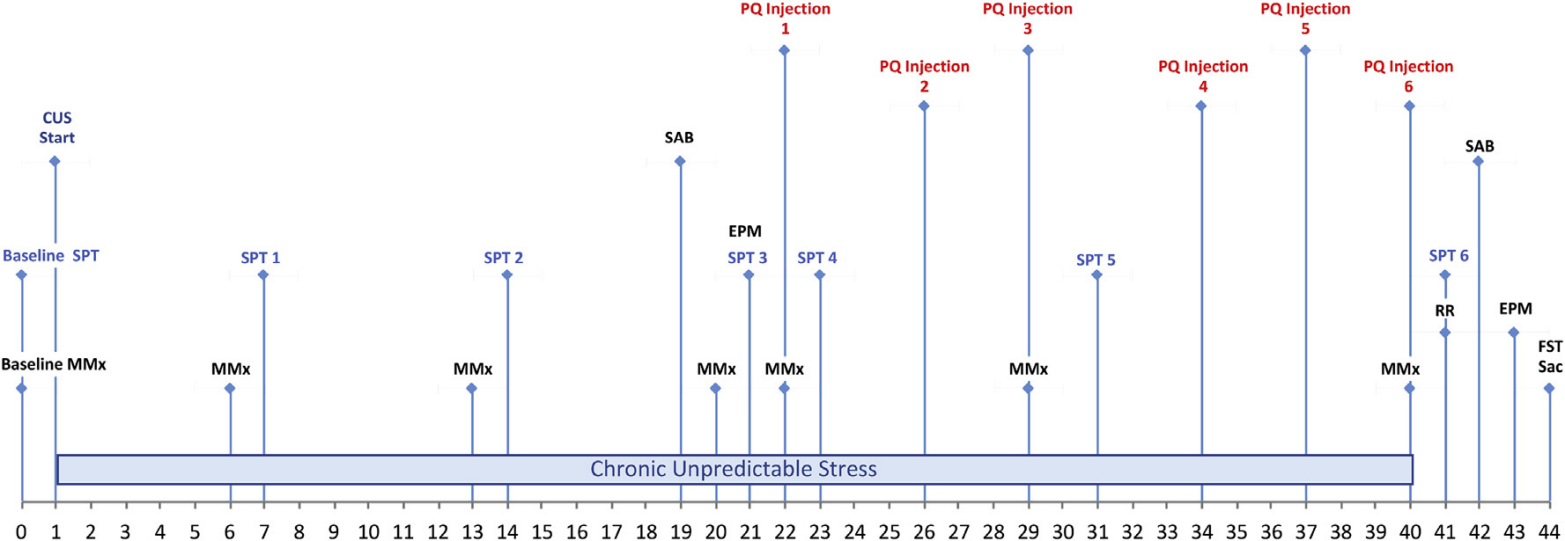
**Schematic timeline of study**. Schematic timeline (CUS = chronic unpredictable stress; EPM = elevated plus maze; FST =forced swim test; MMx = Micromax; PQ = paraquat; RR = rotarod; SAB = spontaneous alternation behavior Y maze; SPT = sucrose preference test).

A schematic timeline for the experiment can be observed in Fig. 1. Fifty-six male C57BL6/J mice were obtained at 3 months of age from Charles River Laboratories Montreal, QC, CAN) and were acclimated to our vivarium for 2 weeks prior to the start of the experiment. All animals were singly housed in standard polypropylene cages (27 × 21 × 14 cm) and maintained on a 12-h light/dark cycle with lights on at 08:00 h. A diet of Ralston Purina (St. Louis, MO) mouse chow and water was provided ad libitum, and room temperature was maintained at ∼21 °C. The Carleton University Committee for Animal Care approved all experimental procedures and complied with the guidelines set out by the Canadian Council for the Use and Care of Animals in Research.

One week prior to the commencement of the study, baseline home cage activity and sucrose preference measures were carried out as described below. Any animals not displaying an initial preference to the 1% sucrose solution were removed from the experiment. Mice (n = 12/group) were then randomly assigned to one of the four experimental conditions (No stress/Saline; No stress/Paraquat; Stress/Saline; Stress/Paraquat). The study occurred over a 6-week period upon which animals received 3 weeks of chronic unpredictable stress followed by saline or paraquat exposure (twice per week for 3 weeks) which occurred while the chronic unpredictable stress or no stress regimen continued. Animals received behavioral tests assessing motor, cognitive, anxiety, and depressive-like characteristics at various time points.

At the end of the experimental paradigm, all animals were either rapidly decapitated or transcardially perfused (i.e. four days following last injection and 5 min after the final behavioral task). All behavioral tests (apart from home cage locomotor activity and sucrose preference testing) were carried out between the hours of 08:00 and 13:00 in order to minimize any diurnal variations.

### 2.2 Chronic unpredictable stress, injection protocol, and behavioral testing

All animals in the experiment were randomly assigned to a 6-week chronic unpredictable stress or a non-stress control group placed in a room separate from stressed mice. Animals in the stress condition received two stressors/day, one in the morning between the hours of 08:00 and 12:00, and one afternoon between the hours of 13:00 and 18:00. Stressors occurred on a variable unpredictable schedule and ranged from mild to severe as per the rationale given by Litteljohn et al. (2014). The chronic variable stressor regimen included the following stressors: overnight exposure to predator (rat) odor which consisted of placing the mouse in a cage containing soiled bedding and rat feces; lights on during total dark phase (i.e. between 20:00 and 08:00); placement of mouse in an empty polypropylene cages (27 × 21 × 14 cm) and exposing them to 5 min of room temperature air from a hair dryer on a low setting; overnight social stress exposure which consisted of placing the mouse in a CD1 retired breeders soiled cage; overnight placement in an empty cage (i.e. void of any nesting or bedding); hanging mouse from tail (1 min); tilting the mouse's cage at a 30° angle (overnight or up to 4 h); 5 min tail pinch which consisted of placing a small insulated clamp over the tail (beginning at the top); 30 min exposure to social stress which consisted of placing the mouse in the cage of a new aggressive retired breeder CD1 mouse (n = 20; Charles River) until socially defeated (defined as submission or first hostile contact by experimental mouse). Upon submission or first contact, a mesh divider was placed between the two animals for the subsequent duration of the session; 30-min restraint in triangular plastic bags with a nose-hole for breathing.

After the first three weeks of the chronic unpredictable stressor procedure, intraperitoneal (i.p) injection of 10 mg/kg paraquat (1,1′-dimethyl-4,4-bipyridinium dichloride; Sigma Aldrich, St Louis, MO, USA) or physiological saline (Sigma Aldrich) commenced. Injections were administered twice a week for 3 weeks on a regular basis between the hours of 08:30 and 09:30. We have used various paraquat doses in the past and the current dosage and injection regimen is based on our previous studies demonstrating a reliable loss of SNc dopaminergic neurons (e.g. Mangano et al., 2011; Rudyk et al., 2015). On injection days, during the 30 min immediately preceding each injection, the stressed animals were either socially defeated or physically restrained on an alternating fixed schedule. All mice continued to have home cage locomotor activity testing, as well as sucrose preference testing during this period. At the end of the stress and injection period, behavioral tests were conducted for all mice to evaluate signs of: (1) behavioral despair (using the forced swim test), (2) anxiety (using elevated plus maze), (3) cognitive impairment in working memory performance (using the spontaneous alternation behavior Y-maze), (4) total home cage activity, and (5) movement coordination (i.e. using our rotarod system) as described below.

#### 2.2.1 Home cage locomotor activity

Spontaneous home cage locomotor activity was measured over a complete 12 h light/dark cycle using our Micromax (MMx) infrared beam-break apparatus (Accuscan Instruments, Columbus, OH, USA) as previously described (Litteljohn et al., 2010). Following a 30 min acclimation period in our behavioral testing room post nestlet removal, measurements of home cage locomotor activity occurred once at baseline (Day 0), then again at the end of the 1st (Day 6), 2nd (Day 13), and 3rd (Day 20) week in the pre-injection period. At the beginning of the injection phase of our study, home cage locomotor activity measurements were taken the evening of the 1st (Day 22), 3rd (Day 29) and 6th (Day 40) injection. This behavioral test occurred at least 3 h following the variable afternoon stressor.

#### 2.2.2 Sucrose preference test

In order to assess whether or not paraquat exposure is associated with depressive-like behaviours, and whether or not chronic unpredictable stress alters this behavioral outcome, a modified sucrose preference test was conducted (Rudyk et al., 2017; Willner et al., 1987). All animals received sucrose preference training for a period of 5 days upon which they received 2 days of 2% sucrose solution followed by 3 days of a 1% sucrose solution post acclimation. Baseline measurements were taken on the 5th day of training. On baseline and testing days, animals were simultaneously exposed to two 200 ml bottles containing 1% sucrose solution or tap water randomly placed approximately 1 cm apart from each other. Amount of solution drank was determined based on bottle weights before and 12 h after placement (i.e. over a complete 12-h light/dark cycle). Preference for the sucrose solution was calculated according to the following formula: sucrose intake/(sucrose intake + water intake)*100. In the pre-injection phase of our study (i.e. the first three weeks) non-stressed and stressed mice received the sucrose preference testing at the end of the 1st (Day 7), 2nd (Day 14) and 3rd (Day 21) week. In the injection phase of our study animals received sucrose preference testing one day after the 1st (Day 23), 3rd (Day 30), and 6th (Day 41) injection to omit the possibility of any sickness behaviours. This behavioral test occurred at least 3 h following the variable afternoon stressor.

#### 2.2.3 Spontaneous alternation behavior Y-maze

In order to assess working memory function is induced by chronic paraquat exposure similar to chronic unpredictable stress exposure, and to assess whether or not chronic unpredictable stress alters this performance, an adapted version of the Y-maze was used (Wall and Messier, 2001). The Y-maze consisted of three arms each 40 cm long X 3 cm wide enclosed by 13 cm high walls which converged on an equilateral triangle at the centre. Each animal was individually placed at random in one of three enclosed arms for a total of 8 min. Alternate arm returns (AAR), same arm returns (SAR), and spontaneous alternation behavior (SAB) performance was recorded when an animal had placed all 4 paws in the arm runway outside of the centre triangle. SAR's were defined as when an animal left a previously entered arm and then returned to the same arm, with at least 2 paw entry into the centre triangle and without total entry into another arm. AAR was defined as when an animal returned to a previous entered arm after 4 paw entry into another arm (for example arm A to B and then back to arm A). SAB performance was defined when an animal had entered each arm with 4 paws in a sequential order without returning to a previous arm (for example arm A to B followed by arm B to C).

For data analysis, the following equations were used: %SAR = total number of same arm returns/total number of arm entries X 100, %AAR = alternate arm returns/total arm entries X 100, and %SAB = total number of sequential alternations/total arm entries X 100. The Y-maze was given twice to mice applied to the stressor condition (i.e. once after exposure to three weeks of chronic unpredictable stress and before exposure to saline or paraquat (Day 19), and then again three days following the last injection (Day 42). Performance in this test was assessed only three days following the last injection (Day 42) in animals assigned to the non-stressed condition.

#### 2.2.4 Elevated plus maze

In order to measure whether or not paraquat induce anxiety-like behavior, and whether or not chronic stress alters performance in this task, the elevated plus maze was used, as previously described (Salmaso et al., 2016). The elevated plus maze consists of 4 arms (24.8 cm long X 7 cm wide) with two closed arms enclosed by 21 cm high walls and elevated approximately 60 cm off the surface of the floor. Each animal was individually placed in the centre of the four-arm maze, and behavior was recorded for a total of 6 min using our Any-Maze software, version 4.71. Anxiety-like behaviours measured included the percent time spent in the open versus closed arms during the last 5 min of the test. The maze was cleaned with 10% EtOH between trials. Anxiety performance in the elevated plus maze was measured twice in mice applied to the stressor condition (i.e. once after exposure to three weeks of chronic unpredictable stress (Day 21) and then again three days following the last saline or paraquat injection (Day 43).

#### 2.2.5 Rotarod

In order to assess whether or not paraquat exposure affects motor coordination and balance, and whether or not chronic unpredictable stress exacerbates these effects, the rotarod task was used (AccuRotor, AccuScan Instruments, Columbus, OH). Our rotarod protocol consisted of a total of three days in which the animal is exposed to a motorized rod (1 cm in diameter) encased in a test chamber (44.5  cm × 14  cm x 51 cm; Accuscan Instruments) a total of three times (spaced 1 h apart from each other to minimize fatigue) each day for up to 5 min. On the first two days (considered training days) the animal was exposed to a rotating rod which maintained a constant speed (12 rpm for day 1, and 22 rpm for day 2) for a total of 5 min whereby they were quickly placed back on the rod when contact with the rotating drum was not maintained. On the third day (considered testing day), the rotating rod increased gradually from 4 to 44 rpm over the 5-min time span and the time the animal spent on the rotating rod during each trial was measured. In adherence with previous study protocols, the lowest amount time an animal spent on the rotating rod was removed and the remaining times were averaged, presumably to eliminate any accidental slips. The rotarod test was carried out one day after the last injection (Day 41). The chambers were cleaned with 10% EtOH between trials.

#### 2.2.6 Forced swim test

The forced swim test (FST) was conducted in order to assess whether the depressive-like effects (i.e. behavioral despair) that are typically induced by chronic unpredictable stress exposure might be similar to those induced by our paraquat dose regimen, and to assess whether or not chronic unpredictable stress alters the altered behavior provoked by the toxin. A modified version of Porsolt et al. (1977) FST was used whereby mice were individually placed in a glass cylinder 20 cm in diameter that contained temperature controlled water (22 ± 1 °C) at a depth of approximately 20 cm for a total of 6 min. Time immobile was recorded on our camera and scored by an independent observer blind to all experimental conditions. Immediately following the task, animals were dried off and placed in their home cage and quickly transferred to necropsy where rapid decapitation or perfusion was performed. This test occurred four days following the last chronic unpredictable stress or saline or paraquat injection (Day 42).

### 2.3 Brain extraction

Five minutes following the final behavioral task (between 8:30am and 11:00am), mice were either rapidly decapitated (n = 6/group) or transcardially perfused (n = 6/group) and tissue was collected for Western blot or immunostaining respectively. For rapidly decapitated animals, a chilled micro-dissecting block that contained slots (0.5 mm apart) for single edged razor blades was used. Brains were quickly excised and the hippocampus was micro-punched from coronal brain sections to assess the effects of paraquat exposure and combined chronic unpredictable stress and paraquat on these regions. The tissue was immediately frozen upon dissection and stored at −80 °C until processing. In transcardially perfused mice, all animals were given an overdose of sodium pentobarbital and transcardially perfused with 5 ml 0.9% saline (pH 7.2) followed by approximately 45 ml of 4% paraformaldehyde (PFA). Brains were excised and post-fixed at 4 °C for 24 h in 4% PFA. The following day brains were placed in a 10% sucrose 0.1 M phosphate buffered (PB) solution (pH 7.4) two times 6 h apart from each other and stored at 4 °C between washes. Following these washes, brains were placed in a 30% sucrose PB solution for 48 h and then flash frozen at −80 °C until processing.

#### 2.3.1 Immunostaining

Immunohistochemistry was performed to assess whether paraquat, chronic unpredictable stress exposure or the Stress × Paraquat interaction influenced: (1) microglia activation in the SNc, (2) the loss of dopamine fibers in the stratum or (3) dopamine cells in the SNc. As such, markers for microglia (i.e. ionized calcium-binding adapter molecule 1; IBA1) and dopamine (i.e. tyrosine hydroxylase; TH) were selected. IBA1 is a membrane bound protein specifically expressed on microglia/macrophages and is highly upregulated in activated glial cells. As such, visual expression of the protein can help distinguish between activated and non-activated states (Ito et al., 1998). TH is the rate limiting enzyme responsible for dopamine production, and can thus be used as an effective dopamine cell marker in the SNc and striatum respectively.

Brains were sliced (40 μm thick for striatum; 40 μm thick for SNc) on our Shandon AS620 cryostat (Fisher Scientific, Ottawa, ON) and sections were immediately placed in a 0.1 M PB solution containing 0.1% sodium azide (pH 7.4). Every second section was selected for each stain (i.e. striatum TH; SNc TH; SNc IBA1). For TH staining, upon tissue processing, slices were washed in a phosphate buffer saline (PBS) (pH 7.4) three times for a period of 5 min each, followed by a 30-min incubation in 0.3% hydrogen peroxide in PBS. Slices were then washed in PBS three times 5 min each and a 1-h incubation in blocking solution containing 5% normal goat serum (NGS), 0.3% triton-X, with 0.1 M PBS, pH 7.2 commenced. Following removal of the blocker, slices were then incubated in primary antibodies (anti-mouse TH 1:2000; ImmunoStar). The following day, primary antibody removal occurred as sections were washed in PBS three times for a period of 5 min each. Following the washes, secondary antibodies were applied to striatum (anti-mouse IgG; 1:500) for a period of 2 h, and to SNc (anti-mouse HRP; 1:200) sections for a period of 4 h. Following a three X 5-min wash striatum sections were incubated in HRP (1:1000) for an additional 2 h. All sections were then washed in PBS three times 5 min each and sequentially exposed to a DAB reaction containing 30% hydrogen peroxide for visualization of HRP. Sections were washed in PBS three times 5 min each following DAB exposure. All sections were then slide mounted and set to dry overnight. Sections were then dehydrated using a series of alcohol and clearene washes and sequentially cover-slipped using DPX. All incubations occurred at room temperature.

For IBA1, upon tissue processing, slices containing the SNc were washed in PBS (pH 7.2) three times for a period of 5 min each, followed by a 1-h incubation in blocking solution containing 5% normal goat serum (NGS), 0.3% triton-X, with 0.1 M PBS (pH 7.2). Following removal of the blocker, slices were then placed in anti-rabbit IBA1(Abcam) at a dilution of 1:1000 in a primary antibody solution containing 5% NGS, 0.3% Triton X, 0.3% BSA, 94.4% PBS for a period of 2 h. Sections were then washed in PBS three times for a period of 5 min each and then reacted with a secondary Alexa 594 antibody for the appropriate species at a dilution of 1:1000 in a solution also containing 5% NGS, 0.3% Triton X, 0.3% BSA, 94.4% PBS. In order to get a representative picture of the SNc slices were also stained with anti-mouse TH (1:1000) in the same primary solution followed by a secondary Alexa 350 antibody following appropriate washes. The signals were visualized with immunofluorescence confocal microscopy using Zeiss image acquisition software (Zeiss LSM 510). All slices were the same distance from bregma.

#### 2.3.2 Microglia quantification

State of microglia cell activation of IBA1 sections in the striatum and SNc was rated using a scale describe previously (Bobyn et al., 2012). Activation state of microglia was scored on a scale of 0 (non-activated) to 2 (highly activated). A score of 0 was given when all microglia were in their quiescent state upon which these cells have highly ramified processes that are actively surveying the microbial environment of the brain parenchyma. A score of 1 was applied when microglia were in an intermediary (moderate) phase of activation upon which less than 10 cells displayed this state. A score of 2 was applied when the majority of cells were in an active state defined as having an amoeboid like shape with little to no ramified processes. An observer blind to all experimental conditions scored all sections.

#### 2.3.3 Quantification of SNc TH-positive neurons

In order to assess the number of dopamine producing neurons in the SNc, stereological procedures were conducted as previously described (Mangano et al., 2011). SNc TH + serial sections were quantified between bregma levels −3.08, −3.16, −3.28, −3.40, and −3.52 by an observer blind to all experimental conditions. Cell counts were conducted using our Micro Bright Field Inc. Stereo Investigator software upon which the entire SNc was outlined under a 2.5x magnification and counts were completed using a 60x oil immersion objective lens.

#### 2.3.4 Quantification of striatal TH-positive neurons

In order to number of TH + terminals in the striatum, densitometry measures from photopictomicropraphs were used as described previously (Mangano et al., 2011). In brief, for each striatal section, an area is selected under a 10x magnification and background threshold was determined from images converted to an eight-bit format. The total number of white (background) to black (TH+) pixels is determined using Image J software that uses an algorithm used to calculate upper and lower thresholds.

#### 2.3.5 Western blot

Tissue punches collected from hippocampus were used to detect glucocorticoid receptor (GR) and phosphorylated glucocorticoid receptor (pGR) expression, as well as to detect levels of brain derived neurotrophic factor (BDNF) (marker of plasticity) in the hippocampus. Indeed, in the current study, we were interested in paraquats effects outside of the nigrostriatal system, and in particular to determine if paraquat acts similar to a psychological stressor, in addition to evaluating whether chronic stress influences the effects of the toxin. As such, research from our lab suggested that paraquat can cause upregulation of the stress relevant hormone corticosterone, as well as lead to the disruption of hippocampal functioning by altering BDNF levels (Litteljohn et al., 2011; Rudyk et al., 2017).

Whole cell lysates from brain regions collected were homogenized in Radio Immuno Precipitation Assay (RIPA) buffer [50 mM Tris (pH 8.0), 150 mM sodium chloride, 0.1% sodium dodecyl sulphate (SDS), 0.5% sodium deoxycholate and 1% Triton X-100] mixed with 1 tablet of Complete Mini ethylenediaminetetraacetic acid (EDTA)-free protease inhibitor (Roche Diagnostics, Laval, QC) per 10 mL of buffer and then sonicated for 10 s in ice cold water. The lysed cells were then centrifuged at 5000 RPM for 10 min at 4 °C. The supernatant was then extracted and protein concentration was determined using bicinchoninic acid (BCA) method (Thermo Scientific, Cat # 23 227). Following protein concentration determination, supernatant was placed in 5x loading buffer and the protein was denatured when placed in a 5 min heating block at a temperature of 105 °C. Following this step, samples were then placed in a −20 °C freezer until Western blotting commenced.

On day one of analysis, proteins were separated using sodium dodecyl sulphate-polyacrylamide gel electrophoresis (SDS-PAGE). After electrophoresis, proteins were transferred for 1 h at 4 °C at 100 V in transfer buffer solution (25 mM Tris-base, 192 mM Glycine, 20% methanol), onto a polyvinylidene difluoride (PVDF) membrane (Bio-Rad, Cat#162–0177). Thereafter, membranes were dried overnight. The following day, membranes were reactivated using methanol and total protein load concentration was determined.

In order to determine total protein, after a brief methanol rinse (5 s), membranes were incubated in REVERT total protein solution for a period of 5 min followed by placement into a REVERT wash solution (6.7% Glacial Acetic Acid, 30% Methanol, in water) two times 2 min each. Membranes were then quickly rinsed with distilled water and imaged on our LI-COR Odyssey imaging system on the 700 channel for an exposure period of 2 min. Membranes were then rinsed immediately post imaging in tris buffer are then were blocked with 0.5% fish gelatin in TBS for 90 min. Membranes were incubated with mouse anti-BDNF (1:1000), rabbit anti-GR primary antibody (1:500; Sigma), rabbit anti-phosphorylated-GR (1:1000; Cell Signaling Technology), for a period of 60 min in 0.05% fish gelatin in TBS with 0.1% tween. Any unbound antibody was removed using 15 mL of TBS-T/membrane at room temperature. Membranes were then incubated for 1 h in infrared conjugate directed against the species the primary antibody was raised in (mouse, rabbit, rat 800, LI-COR) at a concentration of 1:20 000 in 0.5% fish gelatin solution containing 0.2% tween and 0.01%SDS. Membranes were then washed in TBS-T (4 × 5 min) followed by 2 × 5 min washes in TBS and read on our Licor Odyssy system at the appropriate wavelength for 6 min.

#### 2.3.6 Corticosterone assay

In order to test for differences in corticosterone levels an ELISA corticosterone determination assay (Corticosterone #900–097, Lot# D1260724) was performed using trunk blood collected immediately after decapitation. Briefly, all blood samples were collected in EDTA coated Eppendorf tubes, after which they were spun for 20 min at 4 °C (20 000 g). After serum collection, samples were immediately frozen and stored at −80 °C. Corticosterone determination was then performed using our microplate reader and quantified.

### 2.4 Statistics

All data were analyzed by a 2 (Stress; no stress vs. Stress) X2 (Injection; saline vs. paraquat) analysis of variance (ANOVA) followed by Fisher's Least Significant Difference (LSD) test (p < 0.05) where appropriate. When assessing the effects of the first three weeks of chronic unpredictable stress exposure data was analyzed using an Unpaired Student's *t*-Test. Additionally, analysis of the sucrose preference test and total home cage locomotor activity, was completed using appropriate repeated measures ANOVA's conducted with *Time* as the 3rd independent variable followed by the relevant posthoc analysis using Fisher's LSD. All data was analyzed using the statistical software SPSS (2015) and differences were considered statistically significant when *p* < 0.05.

## 3 Results

### 3.1 Effects of first three weeks of chronic unpredictable stress exposure

**Fig. 2:**
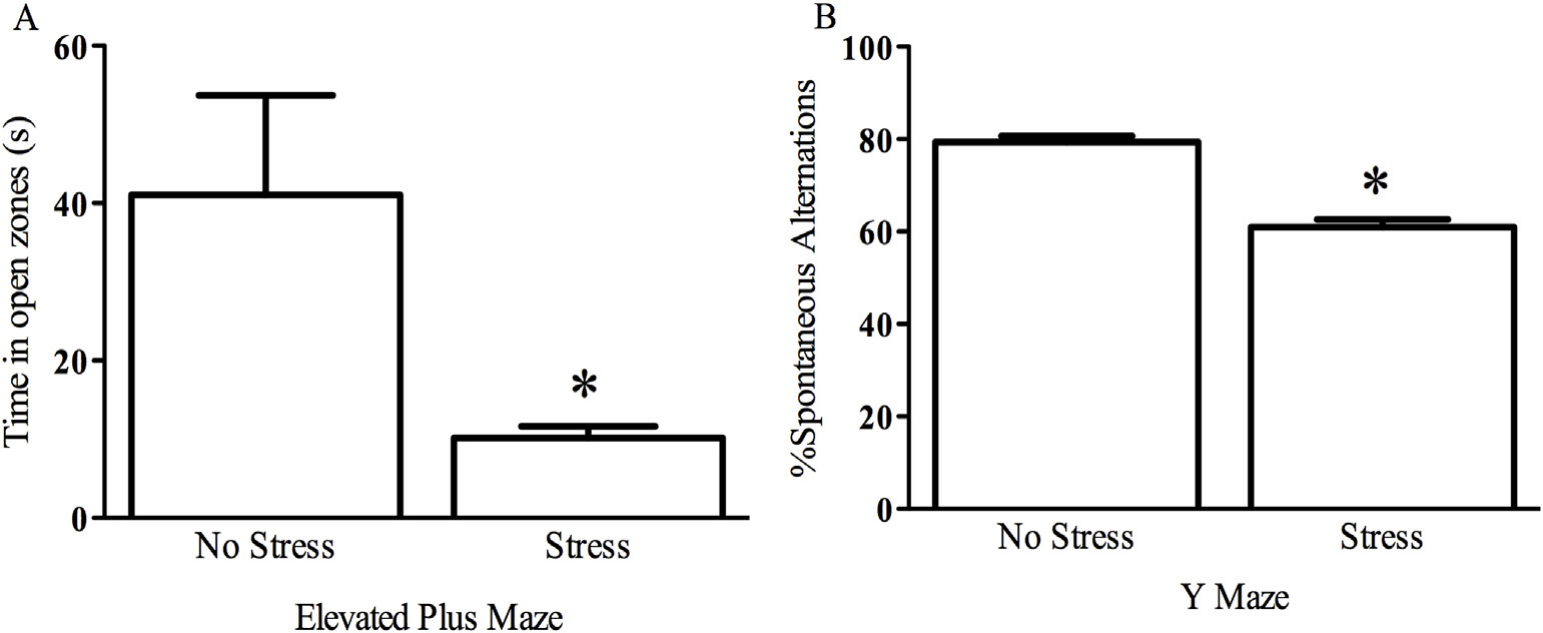
**Three weeks of chronic unpredictable stress induces anxiety-like behavior and working memory deficits**. Mice that received three weeks of chronic unpredictable stress spent significantly less time in the open zones of the elevated plus maze (panel A) indicating an anxious-like state. Stressed mice also displayed working memory deficits as made evident by reduced overall spontaneous alternations (panel B). *p < 0.05, relative to non-stressed mice. n = 24 stressed mice compared to 8 controls. All data is expressed as mean ± SEM.

Prior to saline or paraquat exposure, mice received three weeks of chronic unpredictable stress and were tested against non-stressed controls in order to verify the ability to the stressor regimen to induce anxiety and cognitive deficits. Hence, these mice were tested using an elevated plus maze and spontaneous alternation behavior Y maze. Accordingly, we report that our three weeks of stress did indeed induce anxiety-like behavior as made evident in the elevated plus maze, wherein stressed mice spent less time in the open arms than non-stressed counterparts (t = 4.116, *p* < 0.01; Fig. 2 panel A). Furthermore, the chronic unpredictable stress also induced working memory deficits, as stressed mice displayed lower spontaneous alternations than their non-stressed counterparts (t = 6.534, *p* < 0.01; Fig. 2 panel B). As such, we utilized this stressor regimen for the rest of the study.

### 3.2 Chronic unpredictable stress does not alter home cage locomotor induced by paraquat exposure

**Fig. 3:**
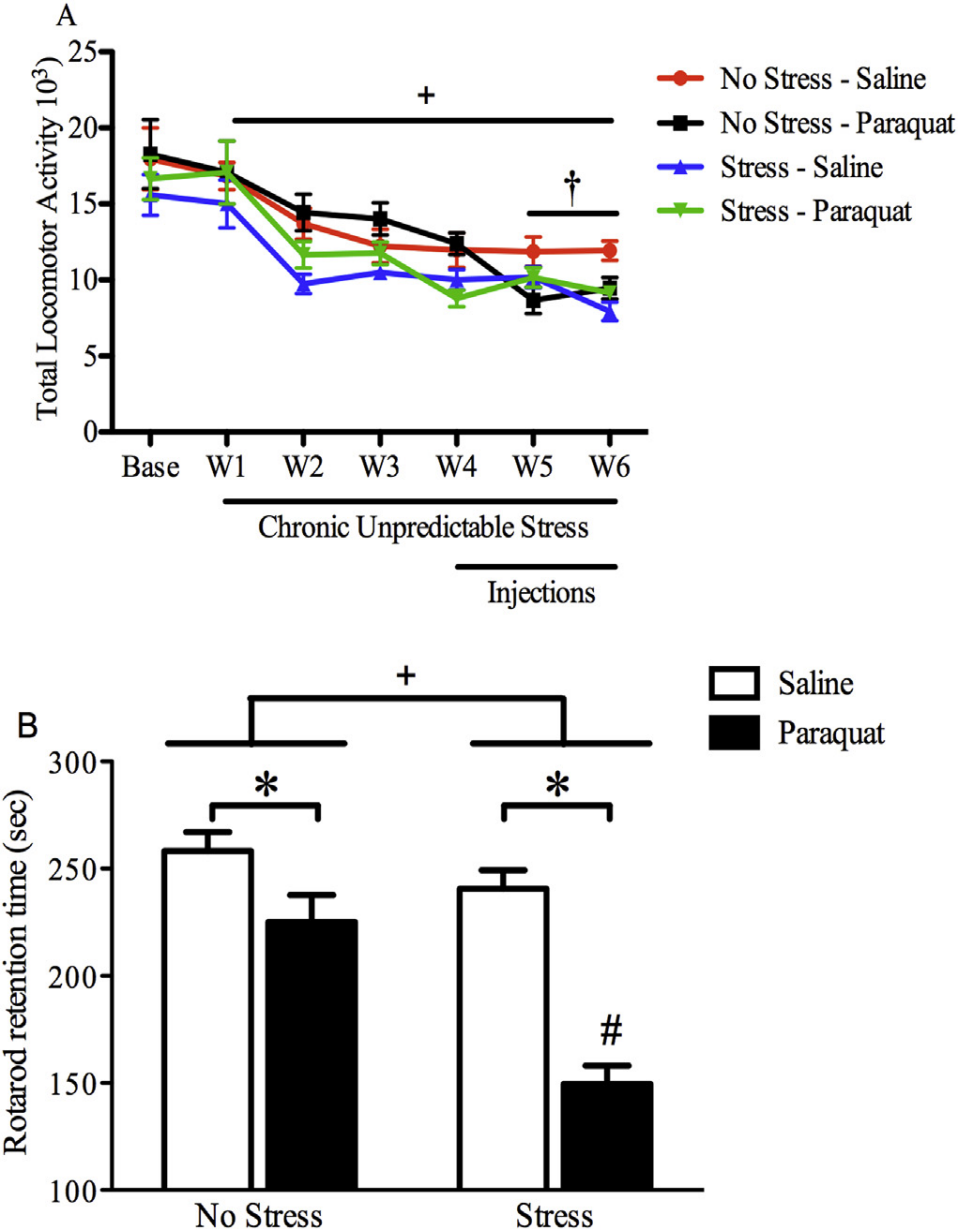
**Chronic unpredictable stress enhanced motor impairment in paraquat exposed mice**. Mice were given three weeks of chronic unpredictable stress followed by paraquat (10 mg/kg; ip) or saline injections twice/week over three weeks upon which the stressor regimen continued. As shown in panel A, stressed mice had a significant reduction in home-cage activity beginning at Week 2 which continued until the end of the study which is depicted by the top horizontal line (n = 11 non-stressed saline mice, n = 11 non-stressed paraquat mice, n = 11 stressed saline, n = 11 stressed paraquat mice). Additionally, paraquat alone provoked a significant reduction in motor activity by Week 5 relative to non-stressed counterparts, however the stress did not enhance motor deficits provoked by the toxin. Panel B shows that both paraquat and stress exposure alone modestly reduced rotarod retention time. However, the combination of the chronic stress and paraquat exposure produced the most robust coordination deficit, that differed from all other groups (n = 12/group). + *p* < 0.05 relative to non-stressed mice irrespective of saline or paraquat exposure; #*p* < 0.05 relative to all other groups; †*p* < 0.05 relative to non-stressed saline treated mice irrespective of stress exposure. All data is expressed as mean ± SEM, post-hoc tests were analyzed using Fisher's LSD.

As depicted in Fig. 3 panel A, mice did not differ in home cage locomotor activity at baseline prior to receiving stressor treatment or experimental injections. However, while it is clear that all mice had a reduction in home cage locomotor activity over time (F (6,234) = 34.95, *p* < 0.001), there was a significant main effect of stress (F (1,39 = 6.606, *p* < 0.05) such that stressed mice displayed lower locomotor activity that began after the first two weeks of chronic unpredictable stress exposure and continued until the final test. Additionally, beginning after the third injection, (Week 5) non-stressed saline exposed mice had the highest levels of locomotor activity relative to all other groups as a significant Stress × Treatment interaction was observed (F (1,43) = 3.902, *p* = 0.05) which was also evident at the 6th injection (F (1,42) = 10.007, *p* < 0.05) and confirmed by post hoc comparisons (*p* < 0.05).

### 3.3 Stress and paraquat treatments synergistically impaired motor coordination

The Rotarod measure of coordinated activity revealed a significant Stress × Injection interaction (F (1, 44) = 8.642, *p* < 0.01). Indeed, as shown in Fig. 3 panel B, the retention time on the rotating drum was significantly reduced by the individual paraquat and stress treatments (p < 0.05, relative to controls), but the combination of these two insults resulted in the greatest reduction, such that levels were lower than all other groups *(p* < 0.05).

### 3.4 Paraquat provoked SNc dopamine cell loss which was not altered by chronic unpredictable stress exposure

**Fig. 4:**
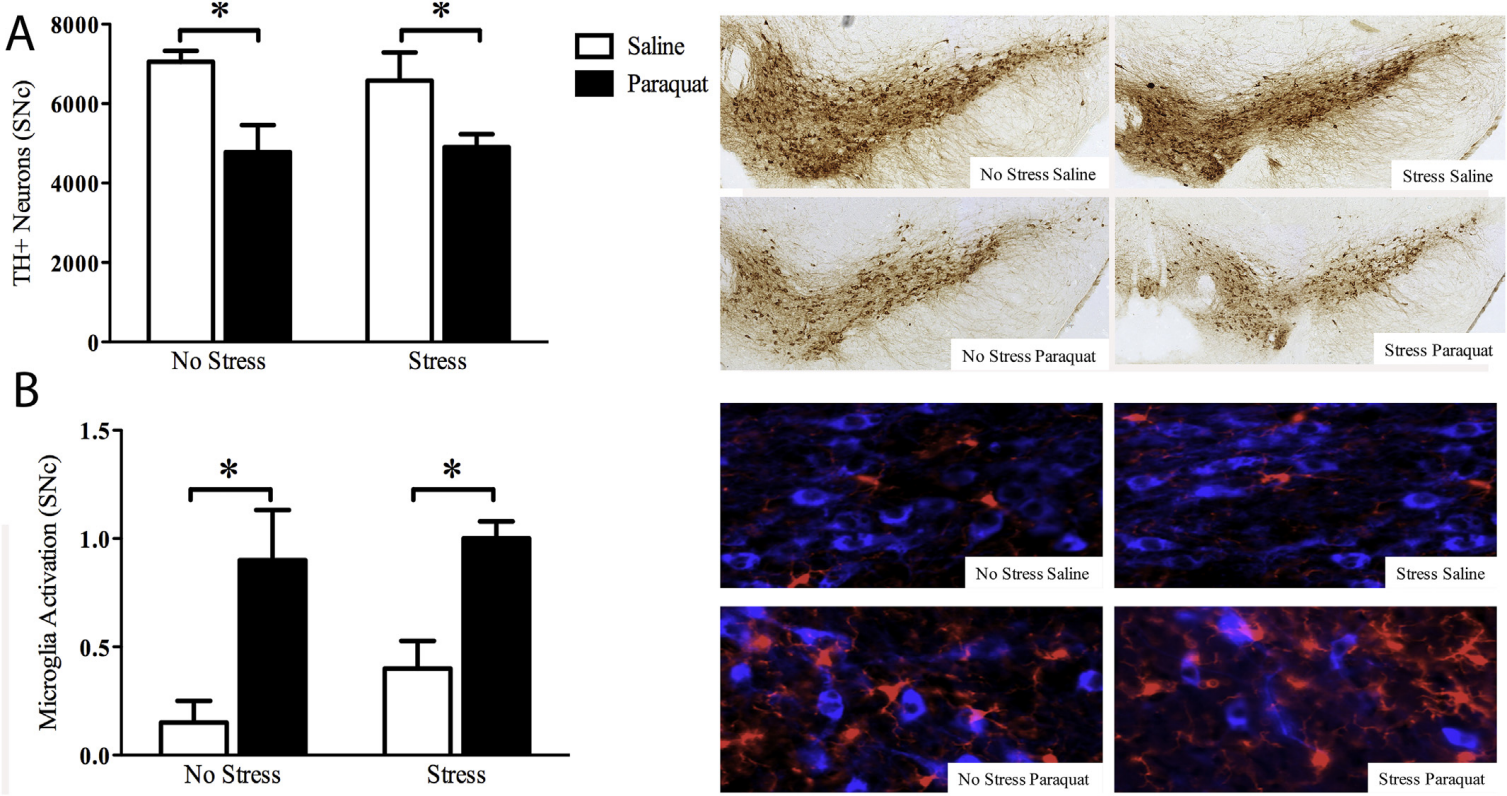
**Chronic unpredictable stress did not alter paraquat provoked SNc neurodegeneration accompanied by regional microglia activation**. As shown in panel A, paraquat induced a significant reduction in TH + SNc dopamine neurons, but this affect was not further influenced by stressor exposure. Similarly, paraquat increased microglial morphological ratings indicative of and activated state, but the stressor had absolutely no effect (panel B). Representative images are shown in the photomicrograph. *p < 0.05 relative to saline treated mice irrespective of stress exposure. n = 5/group. All data is expressed as mean ± SEM.

Four days following the last paraquat injection and stress exposure, mice were sacrificed and a number of surviving TH + dopamine neurons were assessed within the SNc. Regardless of stress exposure, paraquat alone reduced the number of SNc dopamine neurons (F (1,16) = 13.584, *p* < 0.01). This effect was not further altered in mice that received prior and concomitant stress exposure (Fig. 4 panel A).

### 3.5 Paraquat provoked microglia activation in the SNc which was not altered by chronic unpredictable stress exposure

With regards to SNc microglial morphological state, the ANOVA revealed a significant main effect of paraquat (F (1, 16) = 21.130, *p* < 0.01), whereby paraquat exposed animals displayed higher microglia activity. Paralleling the TH + data, stressor exposure had no significant effect. (Fig. 4 panel B).

### 3.6 Chronic unpredictable stress exposure accelerated paraquat induced anhedonia

**Fig. 5:**
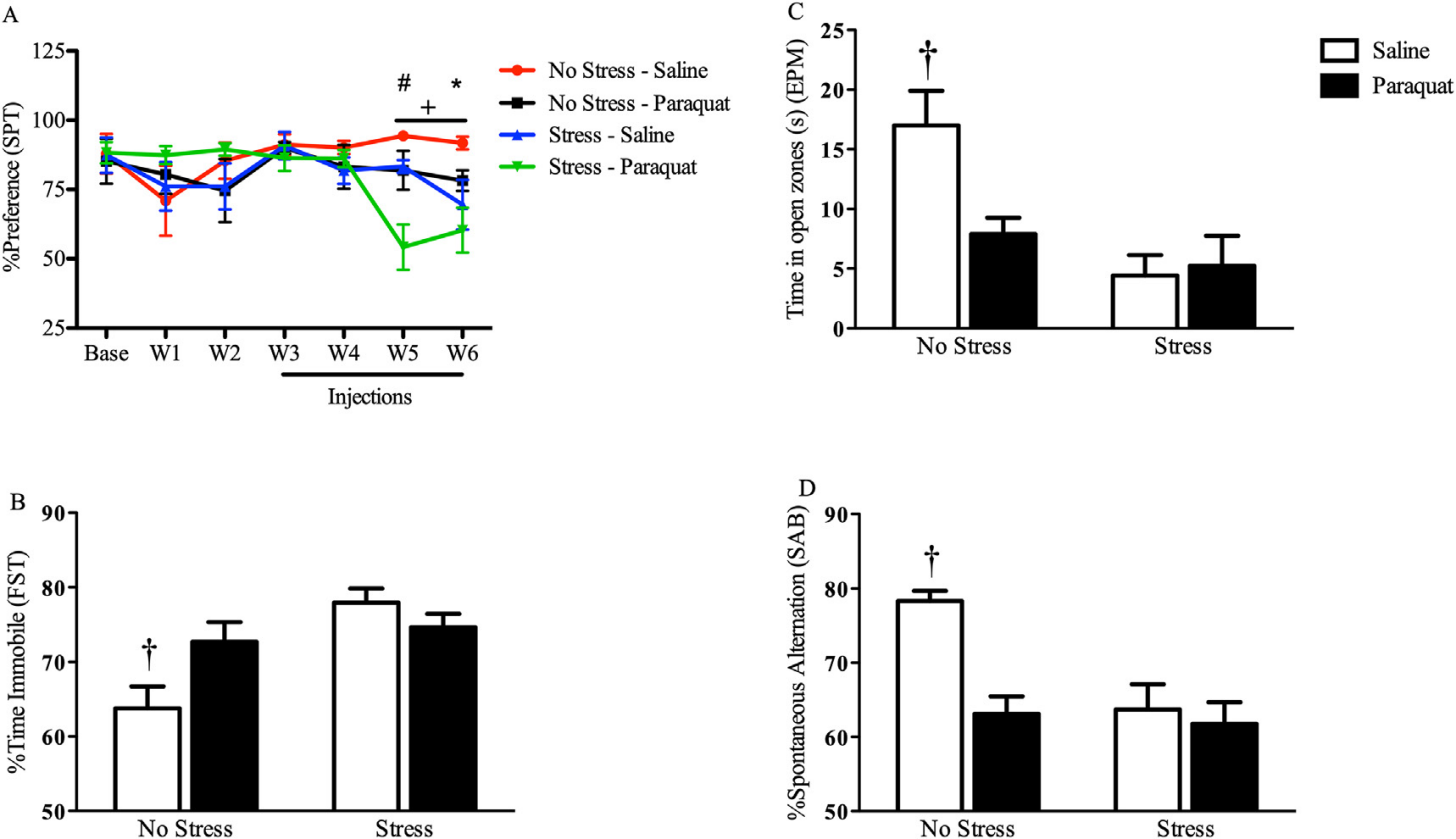
**Paraquat augmented the impact of a stressor on anhedonia (sucrose preference) and provoked behavioral despair, anxiety, and cognitive-like deficits similar to a chronic unpredictable stressor regimen**. As shown in panel A, beginning on Week 5, the individual paraquat and stress treatments significantly reduced sucrose preference. Moreover, the combination of paraquat in mice that had also been receiving the chronic stress exposure produced a further robust reduction in sucrose preference that was significantly greater than all other groups. Panel B reveals that the individual paraquat and stressor treatments each significantly increased the time spent immobile in a forced swim test (n = 12 non-stressed saline mice, n = 11 non-stressed paraquat mice, n = 12 stressed saline, n = 12 stressed paraquat mice). However, there appeared to be no interaction between these two treatments on this behavioral outcome. Similarly, panels C and D, show that paraquat and chronic stressor treatments each reduced time in open zone of an elevated plus maze (n = 12/group) and number of spontaneous alternations in a Y-maze, respectively. But there was no evidence of an interaction between the two treatments, + p < 0.05 relative to non-stressed saline treated animals, †*p* < 0.05, relative to all other groups. All data is expressed as mean ± SEM, post-hoc tests were analyzed using Fisher's LSD.

A sucrose preference test (SPT) was used throughout the 6-week study in order to measure whether or not paraquat-treated animals develop anhedonic symptoms akin to what is expected with chronic unpredictable stress. It was clear that no significant differences were observed in sucrose preference in any of the groups at baseline and during the first four weeks of testing. However, the results indicate a significant Time × Stress interaction (F (6,228) = 2.516, *p* < 0.05), such that at Week 5 stressed animals showed reduced sucrose preference relative to non-stressed animals, however, as evident in Fig. 5 panel A, this effect was specific to stressed mice who were also exposed to paraquat (*p* < 0.05). At week 6 stressed animals exposure to saline or paraquat and non-stressed paraquat animals showed reduced sucrose preference) relative to their non-stressed saline-treated counterparts, and stress did not further exacerbate paraquats effects at this time point (*p* < 0.05).

### 3.7 Chronic unpredictable stress does not alter paraquat induced forced swim deficits

The forced swim test (FST) was administered to assess the amount of time the animals spent immobile, as an indicator of behavioral despair. There was a significant interaction between the stress and paraquat treatments with respect to time spent immobile (F (1,43) = 6.770, *p* < 0.05). The follow up comparisons revealed that both the stressor and paraquat treatments alone elevated immobility time relative to non-stressed saline treated controls (*p* < 0.05), however when these two insults were combined there was no further change in immobility (Fig. 5 panel B).

### 3.8 Paraquat exposure induces anxiety-like characteristics similar to chronic unpredictable stress exposure in the elevated plus maze

As depicted in Fig. 5 panel C, there was a significant Stress X Paraquat Treatment interaction with regard to time spent in open arms of the elevated plus maze (F (1,44) = 5.037, *p* < 0.05). Specifically, paraquat treated animals had reduced time spent in the open arms in the non-stressed animals (*p* < 0.05), but had no effect in the stressed mice. However, the stress treatment itself reduced open arm time relative to the non-stressed saline treated controls (*p* < 0.05).

### 3.9 Paraquat exposure induces cognitive-like characteristics similar to chronic unpredictable stress exposure in the spontaneous alternation behavior Y-Maze

In order to assess whether paraquat exposure induced cognitive-like deficits similar to those brought on by chronic unpredictable stress, and to assess whether chronic unpredictable stress exposure impacts paraquats actions, we assessed working memory in the spontaneous alternation behavior (SAB) Y-Maze. Accordingly, as is clear in Fig. 5 panel D, a significant interaction was observed (F (1,41) = 6.406, *p* < 0.05) such that non-stressed saline exposed mice had a greater number of spontaneous alternations (and thus working memory activity) relative to all other groups (*p* < 0.05). Or alternatively, paraquat induced working memory impairment in this task similar to chronic unpredictable stress and these effects were not altered if paraquat treated mice were also exposed to chronic unpredictable stress regimen.

### 3.10 Paraquat increases plasma corticosterone concentrations similar to a chronic unpredictable stress regimen

**Fig. 6:**
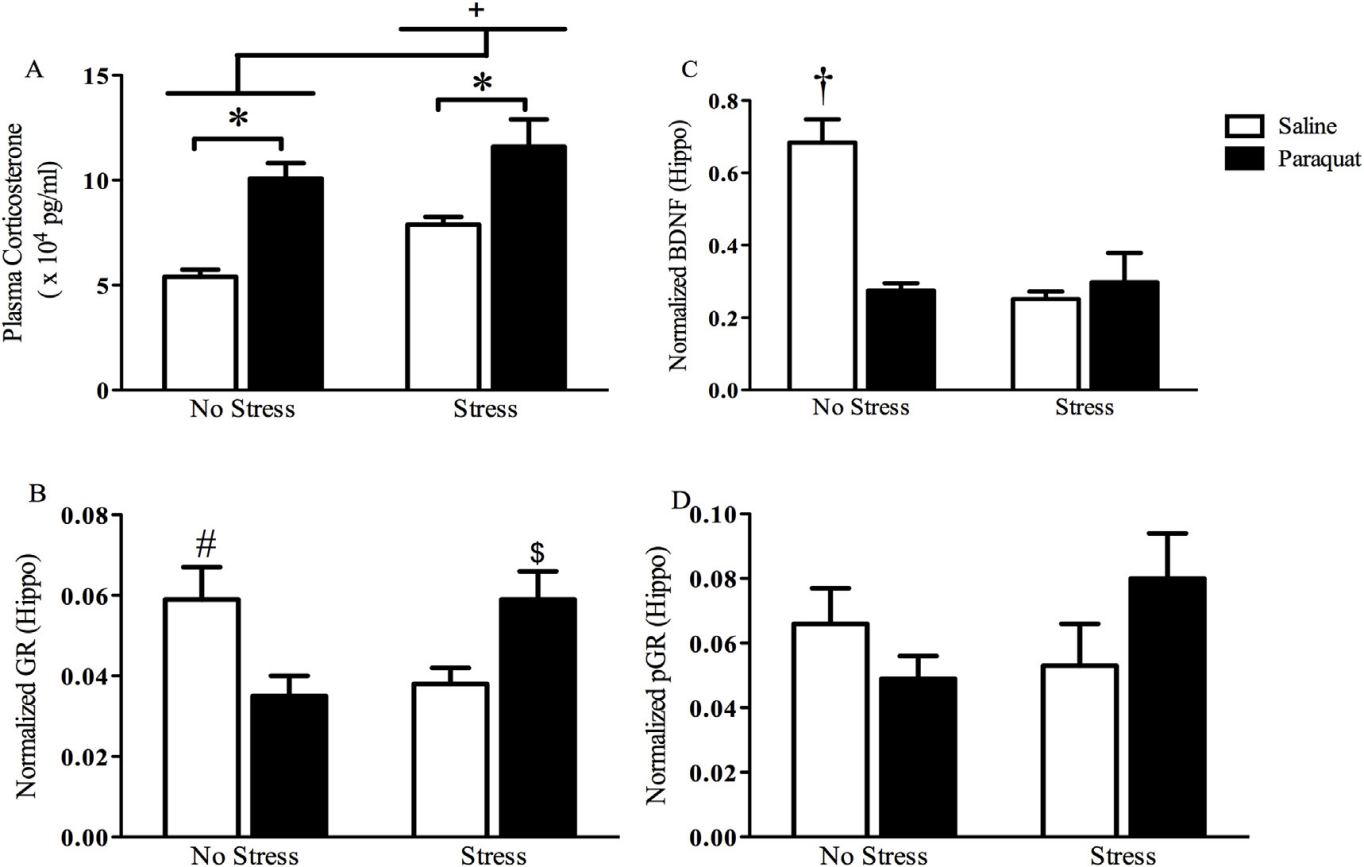
**Paraquat and stressor treatment increased corticosterone and provoked hippocampal alterations in BDNF and GR levels**. As shown in panel A, paraquat and chronic unpredictable stress individually significantly increased plasma corticosterone levels, but there was no interaction evident between the two (panel A; n = 6/group). With regards to BDNF, paraquat and the stressor regimen each reduced hippocampal BDNF levels, but again there was no indication of an interaction between the insults (panel C; n = 5/group). There was however, a significant interaction for hippocampal glucocorticoid (GR) levels, such that paraquat and the stressor individually reduced levels; whereas the combination of paraquat on top of the stressor regimen actually produced GR levels comparable to control animals (panel B, n = 4/group). However, the phosphorylated form of GR (pGR) was not significantly altered by the treatments (panel D; n = 4/group). *p < 0.05 relative to saline treated mice irrespective of stress exposure; + *p* < 0.05 relative to non-stressed mice irrespective of paraquat exposure; †*p* < 0.05 relative to all other groups; # and $ *p* < 0.05 relative to non-stressed paraquat treated mice and saline treated mice exposed to chronic unpredictable stress. All data is expressed as mean ± SEM, post-hoc tests were analyzed using Fisher's LSD.

As shown in Fig. 6 panel A, paraquat treatment influenced plasma corticosterone levels (F (1,20) = 26.052, *p* < 0 0.0001), such that paraquat exposed mice had elevated HPA activity regardless of stress exposure. Stressed animals also displayed elevated corticosterone levels irrespective of paraquat exposure (F (1,20) = 5.896, *p* < 0.05). However, stress did not alter paraquat induced corticosterone activity.

### 3.11 Paraquat and chronic unpredictable stress exposure alter hippocampal BDNF levels

Disturbances in hippocampal plasticity mediated by neurotrophic factors may be important for the co-morbid neuropsychiatric symptoms (i.e. anxiety and depression), as well as the cognitive deficits observed in the disease, and may provide further validation that paraquat behaves similar to a chronic stress exposure which typically results in trophic factor reduction. To this end, we tested hippocampal levels of the neurotrophin BDNF. We found that stress (F (1,16) = 14.519, *p* < 0.05) irrespective of paraquat exposure and paraquat exposure (F (1,16) = 11.280, *p* < 0.05) irrespective of stress exposure significantly reduced BDNF levels relative to saline or non-stress treated mice respectively (Fig. 6 panel C). Likewise, we also found a significant interaction (F (1,16) = 17.949, *p* < 0.05) such that saline treated non-stressed mice had higher levels of BDNF relative to all other groups; however, stress exposure did not further alter the paraquat driven reduction in the BDNF levels.

### 3.12 Chronic unpredictable stress reverses paraquat reduction of hippocampal GR levels

A significant interaction was observed in regards to total GR level (F (1,12) = 13.287, *p* < 0.01). Specifically, as shown in Fig. 6 panel B, and confirmed by follow-up comparisons, paraquat reduced hippocampal GR levels in non-stressed mice, relative to saline or stressed-paraquat treated counterparts (*p* < 0.05). Similarly, saline treated stressed mice also displayed reduced hippocampal GR levels in comparison to their non-stressed saline or stressed-paraquat treated counterparts (*p* < 0.05). No effects were observed in regard to pGR expression (Fig. 6 panel D).

## 4 Discussion

Although empirical data are sparse, it is thought that the widespread brain alterations induced by chronic psychological stress may exacerbate the motor and non-motor behavioral features of PD (Hemmerle et al., 2012). Accordingly, we sought to assess whether the PD-linked toxicant, paraquat, might interact with a chronic unpredictable stress regimen to influence PD-like motor and non-motor outcomes. Excitingly, the combined paraquat injections in the context of the chronic stressor regimen did result in an augmented reduction in motor coordination, as measured on a rotarod task. The stressor and paraquat treatments also collectively promoted an enhanced reduction in sucrose preference at one time point (Week 5). Overall however, for the majority of outcomes no significant interactions were apparent between paraquat and the stressor; this included measures of anxiety (elevated plus maze), behavioral despair (forced swim), home-cage activity, cognitive flexibility (Y-maze), neurodegeneration (TH + neuron counts), microglial reactivity, corticosterone and hippocampal BDNF and GR. That said, paraquat itself had many effects that were similar to that of the stressor treatment, which of course is consistent with our proposition that the pesticide can act as a systemic stressor. This of course, raises the possibility that paraquat might not only promote PD-like primary pathology, but also may also produce depressive or possibly anxiety-like outcomes that resemble the co-morbid features of the disease.

Paraquat has been shown to accumulate in areas outside of the motor system including those highly implicated in cognitive and neuropsychiatric disturbances (Prasad et al., 2009; Yin et al., 2011), thereby giving it ready access to influence such processes. Likewise, the toxin was shown to produce alterations in neurotransmitter activity in areas extending throughout the brain (i.e. the prefrontal cortex, hippocampus, locus coereulus, and hypothalamus) (Chanyachukul et al., 2004; Mitra et al., 2011). Similarly, oxidative and inflammatory effects induced by the toxin have also been observed in frontal, limbic, and brain stem regions (Chanyachukul et al., 2004; Mangano et al., 2011; Mitra et al., 2011). In fact, ability of the toxin to induce cell death in brainstem and midbrain structures, including the locus coeruleus and ventral tegmental area (VTA), has been shown with exposure to relatively high doses (Fernagut et al., 2007; Ossowska et al., 2006). In combination with the findings from our current study, these results provide support that exposure to environmental pesticides, including paraquat, have a capacity to contribute to the development of non-motor symptoms often evident in PD, in addition to contributing to the cardinal motor deficits.

As expected based our previous findings (Mangano and Hayley, 2009; Mangano et al., 2011; Litteljohn et al., 2011), paraquat caused a statistically significant loss of substantia nigra dopamine neurons, together with microglial activation. Most importantly however, was that superimposition of the chronic unpredictable stressor regimen did not further influence the neurodegenerative or neuroinflammatory processes provoked by the toxicant. This does not however preclude the possibility that an extended more severe stressor regimen might impact dopaminergic neuronal vulnerability. Yet, our findings are particularly significant in light of the fact that psychological stress is such a persistent feature in a large number of PD patients.

Although scant, there are some previous data showing that paraquat can induce cognitive, emotional or affective behaviours in mice (Campos et al., 2013), but the present data greatly extend these findings. Indeed, we found that paraquat induced depressive measures (i.e. anhedonia, behavioral despair/learned helplessness), along with working memory deficits/cognitive impairment, and anxiety-like behavior as measured in the spontaneous alternation behavior Y maze task, the elevated plus maze, and open field test, respectively (Litteljohn et al., 2008). Yet, it should be underscored that while our current findings are in agreement with some previous studies (Chen et al., 2012; Rudyk et al., 2017) they are inconsistent with previous findings by Campos et al. (2013). Perhaps due to treatment administration, age upon exposure, or inherent species differences. Furthermore, the fact that an anhedonic response was not observed at three weeks after the stressor treatment alone is at odds with some previous studies (Zhao et al., 2016), but not others. For instance, four weeks of chronic unpredictable stress did modestly but significantly reduce sucrose preference in C57BL/6 mice (Zhu et al., 2014) but another study using the same mouse strain only observed differences after 5 weeks of stress (Pothion et al., 2004). This could be due to methodological differences, such as water deprivation or not could possibly explain so of these variations. It is also worth mentioning that all mice were currently single housed and this could have had a mildly stressful impact on our control mice, thereby adding background ‘noise’. We should also mention the possibility that repeated testing could have influenced some of our outcomes. Yet, with an eye towards minimizing test-re-test learning confound we waited three weeks before further testing mice on the elevated plus maze and other behavioral tests.

Intriguingly, while stress did not appear to alter the paraquat induced anxiety or cognitive impairments, the anhedonic response was modestly accelerated towards the end (i.e. 5th and 6th weeks) of the experiment when the pesticide exposure overlapped with that of the stressor. We are the first to show that such an interaction on depressive behavior can occur between a psychological stressor and environmentally relevant PD toxicant. Likewise, our own previous work demonstrated that a chronic social stressor together with paraquat reduced sucrose preference to a great extent then did each of the individual insults (Rudyk et al., 2015). However, in the present paradigm, the stressor exposure commenced well before the paraquat injection started and involved intermittent different stressors; whereas in the previous study a social defeat stressor was imposed just prior to each paraquat injection (Rudyk et al., 2015). Thus, paraquat can conceivably modify the impact of qualitatively different psychological stressors upon anhedonic responses.

The present study revealed for the first time that paraquat and a psychological stressor acted together to impair motor coordination (to an extent that surpassed their individual effects), as revealed using a Rotarod test. This is important given the obvious psychological stress that many PD patients undoubtedly experience. In fact, at least anecdotally, tremor severity has been known to be increased during times of psychological distress. The mechanisms responsible for the augmented Rotarod deficit presently observed are not clear. Indeed, we did not find any augmented neuronal loss or microglial activation with the stressor + paraquat combination. However, we did not assess neurotransmitter levels, which clearly could be linked to the deficit. So, it is possible that neurochemical alterations in this motor learning circuit gave rise to the ability of the stressor to augment the impact of paraquat.

With regard to paraquat toxicity, numerous studies demonstrated that it’s oxidative stress effects negatively affect neuroplasticity in parallel with a variety of behavioral disturbances (Chen et al., 2010; Czerniczyniec et al., 2011; Desplats et al., 2012; Litteljohn et al., 2011; Mangano et al., 2012; Mitra et al., 2011; Songin et al., 2011). Our present findings demonstrated that paraquat reduced BDNF and glucocorticoid receptor (GR) in the hippocampus (Anisman et al., 2008). Similarly, psychological stressors were previously noted to act in this manner and conversely, the administration of the trophic factors, such as BDNF, reversed the neuropsychiatric and cognitive effects induced by a number of stressors (Gourley et al., 2008; Schmidt and Duman, 2010). In addition, the chronic stressor treatment alone as expected (Marin et al., 2011), was found to also provoke a reduction in hippocampal BDNF and had behavioral consequences. However, as already noted there were no synergistic or even additive effects with paraquat and stress co-administration with regards to BDNF levels.

Beyond the BDNF changes, paraquat also activated the HPA axis in a manner similar to that of more traditional stressors. In fact, we found that paraquat alone actually increased levels of corticosterone to a greater extent than did the current chronic stressor exposure. When looking at these insults together, there was a slight additive effect but it was clearly apparent that these two insults do not synergize in their ability to stimulate HPA activity. It would not at all be surprising that paraquat and chronic unpredictable stress activate the HPA through differing mechanisms. At the very least, as first mentioned by Herman and Cullinan (1997), the systemic stress of paraquat is acting more directly on the hypothalamus, whereas the psychological elements of the chronic stressor involved higher order interpretation via the cortex and limbic loops. Another element that should be considered is the fact that it could be the distress (including the well documented sickness response) caused by paraquat that is activating the HPA or alternatively, it might be the oxidative stress known to be induced with the brain that gives rise to this effect. Also, the exceedingly long half life of paraquat in the brain (∼28 days), compared to the periphery (8–36 h) would give the compound amble time to cumulatively activate stress circuitry. Finally, of course, caution should be exercised since corticosterone was only assessed once and stress-paraquat interactive effects might have been evident at earlier time points in the study. It is also possible that the rapidity or duration of the corticoid response could have been altered; thus, we can only speak to the magnitude of the response at endpoint.

In parallel with the corticoid changes, hippocampal GR expression was altered by the two insults. In this regard, both the stressor and paraquat markedly reduced GR levels, but curiously, their combination actually cancelled out this effect. This surprising finding is hard to reconcile. But may be related to the ability of the stressor treatment to alter paraquat metabolism, such that the pesticide's accumulation in the brain is somehow limited. It will be recalled that the stressor regimen commenced 3 weeks prior to the first paraquat injection and this may have resulted in compensatory responses that blunt the impact of paraquat. Of course, from an alternate view, perhaps the paraquat treatment also up-regulated signaling pathways that were antagonist or competed with the stressor effects. In fact, we previously found a somewhat similar interaction between paraquat and age (Rudyk et al., 2017). In this case, low dose paraquat treatment (1/10th of the present does) increased the phosphorylated form of GR within the hippocampus, and simply aging alone (17 months of age) had a similar effect. Curiously, however, when paraquat was combined with this excessive age the individual impact of the two insults was absent (Rudyk et al., 2017).

## 5 Conclusions

Results from the current study showed that there was mostly no additive or synergistic interaction between chronic unpredictable stress exposure and paraquat on motor and most non-motor behavioral outcomes (with the exceptions of the Rotarod and Week 5 for sucrose preference). Most importantly, our study did support the notion that paraquat exposure behaves as a systemic stressor, with regards to HPA activity and hippocampal processes, as well as behavioral outcomes. Hence, it is clear that such toxicant exposure can contribute to not only the primary motor but also the wide range of co-morbid aspects of PD.
